# Understanding how personality traits, experiences, and attitudes shape negative bias toward AI-generated artworks

**DOI:** 10.1038/s41598-024-54294-4

**Published:** 2024-02-19

**Authors:** Simone Grassini, Mika Koivisto

**Affiliations:** 1 Department of Psychosocial Science, University of Bergen, Bergen, Norway; 2 Cognitive and Behavioral Neuroscience Laboratory, University of Stavanger, Stavanger, Norway; 3 Department of Psychology, University of Turku, Turku, Finland

**Keywords:** Psychology, Human behaviour

## Abstract

The study primarily aimed to understand whether individual factors could predict how people perceive and evaluate artworks that are perceived to be produced by AI. Additionally, the study attempted to investigate and confirm the existence of a negative bias toward AI-generated artworks and to reveal possible individual factors predicting such negative bias. A total of 201 participants completed a survey, rating images on liking, perceived positive emotion, and believed human or AI origin. The findings of the study showed that some individual characteristics as creative personal identity and openness to experience personality influence how people perceive the presented artworks in function of their believed source. Participants were unable to consistently distinguish between human and AI-created images. Furthermore, despite generally preferring the AI-generated artworks over human-made ones, the participants displayed a negative bias against AI-generated artworks when subjective perception of source attribution was considered, thus rating as less preferable the artworks perceived more as AI-generated, independently on their true source. Our findings hold potential value for comprehending the acceptability of products generated by AI technology.

## Introduction

The rapid advancement of artificial intelligence (AI) has sparked, especially in recent years, a significant debate about its impact on various industries and the future of work and society^1,2^. The significant rise of artificial intelligence systems in recent technological breakthroughs has led to our era being dubbed the “Age of AI”^3^ and the “Fourth Industrial Revolution”^4^. While AI has already made its presence felt in fields such as education^5,6^, healthcare^7^, banking and finance^8^, retail^9^, and transportation^10^, its use in other fields such as communication and art ^11,12^ has also been on the rise particularly with the release of powerful and user friendly generative AI tools like ChatGPT, developed by OpenAI or the Bard model developed by Google, as well as other tools specialized in text-to-images creation as for example DALL-E or MidJourney^13,14^.

The ongoing debate on the relationship between humans, machines, and art is only a recent development of a discussion that emerged during the first half of the 20th century. Already in 1935 Walter Benjamin examined the impact of technical reproducibility on art in his influential essay “The Work of Art in the Age of Mechanical Reproduction”^15^. Benjamin argued that mechanical reproduction techniques, such as photography and film, fundamentally altered the nature of art by diminishing the unique presence or “aura” of the original artwork. However, differently from the technically produced artworks mentioned by Benjamin, generative AI systems have evolved beyond merely copying existing artworks (e.g., art prints) or reproducing images with human assistance (photography), and they currently possess the capability to create seemingly “unique” (at least from a statistical point of view^16,17^) art pieces.

The concept of machines generating original creative and informative content, including text and artwork, raises concerns regarding the relationship between humans and machines, as well as the future of creativity^18^. Although AI technology has automated certain aspects of the creative process, the resulting output has been often reported to lack true originality^19^. Such limitation can be attributed to AI systems' reliance on input data, which constrains their capacity for innovation and novel expression^20^. Despite this, recent research has shown that AI performs excellently tasks commonly employed to assess human creativity^21^. Furthermore, emerging research demonstrates that AI can engage in authentic artistic creation that transcends the mere imitation of existing styles^22–24^.

AI has not only produced paintings reminiscent of famous artists^3^ but also developed original artistic styles^4^ and art pieces of remarkable quality, that have been purchased for significant prices at auctions^25^. AI has participated in a variety of artistic activities, such as composing original songs^26^ and poetry^27^. Previous research has shown that AI-generated works can be indistinguishable from human-made art^27,28^, and blind tests have revealed that people assign high artistic value to them^29^. Consequently, nowadays individuals increasingly face scenarios akin to the Turing test^30^, where they may struggle to differentiate between AI-generated and human-generated art or music.

Advancement in AI technology raises concerns about unemployment, the extinction of humankind^31–33^, and the erosion of humans' privileged and unique position in the world^34^. A widespread dissemination of AI-generated art has the potential to challenge people's beliefs about human nature and artistic creativity, with potential ethical and social consequences^35^. These perceived threats are likely due to AI's ability to shake humans rooted anthropocentric views. The term Anthropocentrism refers to the perceived precedence of the human beings over other species^36^, and it is a widespread bias often evident even in children, although it is more likely to be culturally shaped than innate^37^. This bias, also referred to as human supremacy or human exceptionalism, influences many aspects of life, such as biological thinking^38^, ecology^39^, animal rights^40^, and in general how humans interact with the environment^41^. Schmitt^42^ suggested that a negative bias towards machines could hinder widespread AI acceptance, particularly in AI-generated activities that have been historically believed to be exclusive domain of humans, and AI art may challenge anthropocentric beliefs even more than other AI activities, which often involve analytical or computational skills. People may be less inclined to accept AI involvement in more human-like tasks^43^ or in products with higher symbolic value^44^. Artistic creativity is often considered a core human feature^22,45,46^, so the emergence of AI art could threaten people's anthropocentric worldview. Speciesism, an idea strongly associated with anthropocentrism^47^, has also been individuated in the literature as a possible obstacle for the acceptance of AI.

As a result of such possible threat to their worldview, people may tend to defend their anthropocentric beliefs by downgrading the artistic value of AI-generated art, following a pattern of behavior observed in the evaluation of other moral contexts^48,49^. Individuals frequently remember, create, and assess information in a manner consistent with their present motivations^50^. Considering that AI art can pose a significant psychological challenge, impacting people's ontological security, individuals are likely to be biased in processing information in this context. In addition, the perception of AI-generated content may be influenced by the biases and expectations of human evaluators, who may attribute more value to human-created works due to their assumptions about the inherent qualities of human creativity, or because humans tend to value objects and product more when they believe that they somehow connect them with other humans via their manual work^51^. In ambiguous situations where stimuli can be interpreted in multiple ways, biased information processing driven by motivation is more likely^52^, and artistic production provides a high degree of such interpretational flexibility. Art experiences are highly subjective, making them susceptible to motivation-driven biases and less measurable attribute, enabling the individuals to exhibit biased emotional responses to AI-generated art without directly violating explicit objectivity and measurable rules. In a recent study^53^ it was found that humans have a strong bias against AI-made artworks, which are perceived as less creative and induce less sense of awe compared to human-made art and that the bias is stronger among individuals with stronger anthropocentric beliefs. The authors proposed that the systematic devaluation of AI-made art is a response to a threatened anthropocentric view that reserves creativity exclusively for humans.

In a likely future where people will encounter more and more AI-generated contents, it is increasingly important to examine how individuals perceive and evaluate these contents and identifying the factors that shape their judgments, including their beliefs about the role of AI in creative processes^54^. Despite the recent development and the large debate generated around AI-generated content, there is still a significant gap in our understanding of how individuals evaluate and perceive such content. It is essential to explore whether individuals can accurately differentiate between content created by humans and those generated by AI technologies and how attribution knowledge (i.e., information about the creators of the content) affects their evaluation and reception of the work. Moreover, despite recent evidences that individual factors may mediate how much an individual likes and trust AI systems^55^, it has not been studied how individual differences, such as personality, relationship with technology, level of education, and cultural background, may mediate the way that people perceive and analyze information that perceived as AI generated.

In the present study, the following primary, pre-registered research question was tested:

RQ1: Do individual factors predict how people positively/negatively perceive and evaluate images that are believed to be produced using artificial intelligence?

In the present study, we focus on factors that relate to personality and personal attitudes that are well established in psychological research or that have shown relationship with how individuals interact with art and technology. These variables that selected a-priori before data collection were the Big-Five personality traits^56^, empathy (empathy quotient)^57^, creative identity (creative self-efficacy and creative personal identity)^58^, relationship with art (art interest)^59^, and relationship with technology (attitudes toward media and technology)^60^.

### H1

Higher levels of empathy (H1a), a stronger creative identity (H1b), and a deeper relationship with art (H1c) will predict a negative bias towards the stimuli that are believed to be AI-generated art. This hypothesis is grounded in the assumption that individuals who highly value human interactions (such as empathetic people) and those with a creative identity may perceive AI-generated art as less authentic or expressive compared to human-created art, leading to a negative bias. Moreover, those with a profound connection to art might view AI art as a challenge to the conventional artistic process and human creativity, further contributing to the negative bias.

### H2

A positive attitude towards technology will predict a relatively more favorable view towards stimuli that are believed to be AI-generated. As people become increasingly accustomed to technology, they may be less skeptical of AI's capabilities and less apprehensive about embracing the technology.

### H3

Openness, as one of the Big-Five personality traits, will have a negative correlation with a positive attitude towards art believed to be AI generated. This is because individuals with high openness tend to exhibit a greater interest in art and possess stronger creative identities, potentially fostering skepticism towards AI-generated art.

Regarding the other four personality traits (conscientiousness, extraversion, agreeableness, and emotional stability), we did not formulate specific predictions but will incorporate them as exploratory (explicitly mentioned as exploratory in the pre-registration) predictors in our analyses.

In addition, both the preliminary and the follow-up data analyses, as outlined in the pre-registered data analysis plan, enabled us to investigate the following exploratory research questions:

RQ2: Are participants able to distinguish human-made from AI-generated images? Are individual factors predicting who is better in distinguishing them?

Previous research has shown that humans may not be able any longer to distinguish human products from the products of AI^27,28^. For the case that this hypothesis would be supported (preliminary analyses), an adjustment to our data analysis strategy was included in the pre-registered data analysis plan, as detailed in the Method section. We did not have any initial assumptions regarding whether individual factors might influence the ability to accurately distinguish between human-made and AI-generated images.

RQ3: Do humans have a negative bias toward AI-generated art?

Recent published literature has shown that a negative bias is commonly reported against AI artworks^53^, therefore it is possible that the same bias will be observed in our study. Please note that the pre-registered research question was developed expecting that such bias toward AI-generated outputs exist and that may be mediated by individual characteristics. However, the present research question was not explicitly mentioned in the pre-registration of the study but considered in the data-analysis plan. Conducting the pre-registered analyses enabled us to assess whether such negative bias is evident in our study. These analyses served to examine the general perception of artworks generated by both humans and AI, and to analyze how the perceived source of the artworks influenced the scores for liking and positive emotions.

## Results

Table 1 presents the descriptive statistics for the Big-Five traits and empathy and Table 2 for the TIPI and the EQ scales. The intercorrelations between the variables are reported in the Supplementary Materials file.

**Table 1:** Descriptive statistics for the Big-Five traits and empathy (n = 201).

	Mean	95% Confidence Interval		SD	Minimum	Maximum
		Lower	Upper			
Extraversion	3.24	3.02	3.47	1.61	1.00	7.00
Agreeableness	5.18	5.01	5.35	1.20	2.00	7.00
Conscientiousness	5.25	5.06	5.44	1.36	1.50	7.00
Emotional stability	4.39	4.17	4.61	1.58	1.00	7.00
Openness	4.95	4.78	5.11	1.21	1.50	7.00
Empathy	41.30	39.36	43.25	13.98	9	77

**Table 2:** Descriptive statistics for the SSCS, MTUAS, and the VAIAK scales (n = 201).

Mean	95% Confidence Interval		SD	Minimum	Maximum
	Lower	Upper			
3.60	3.49	3.70	0.763	1.00	5.00
3.39	3.24	3.55	1.113	1.00	5.00
2.21	2.12	2.30	0.662	1.00	4.67
2.69	2.56	2.83	0.983	1.00	5.00
2.85	2.70	3.00	1.075	1.00	5.00
3.24	3.10	3.38	1.006	1.00	5.00
3.53	3.30	3.77	1.674	1.00	7.00
2.50	2.32	2.67	1.252	1.00	6.25

*MTUAS-A-P* Media and Technology Usage and Attitudes Scale: positive attitude, *MTUAS-A-N* Media and Technology Usage and Attitudes Scale: negative attitude, *MTUAS-A-A* Media and Technology Usage and Attitudes Scale: anxiety, *MTUAS-A-TS* Media and Technology Usage and Attitudes Scale: Task switching.

Paired-samples t-tests examined the difference in the dependent variables between human-generated and AI-generated images. The AI-generated images (M = 59.2, SD = 12.7) were liked more than human-generated images (M = 47.3, SD = 13.1), t(200) = 21.03, *p* < 0.001, d = 1.48. Similarly, The AI-generated images (M = 52.2, SD = 13.4) evoked positive emotions more than human-generated images (M = 42.3, SD = 13.7), t(200) = 20.08, *p* < 0.001, d = 1.42. However, the participants could not discriminate whether the images were generated by humans or AI (0 = *most likely human*, 100 = *most likely AI*). The AI-generated images (M = 41.7, SD = 11.6) were rated with small effect size as more likely to be products of humans than the human-generated images (M = 43.3, SE = 11.8), t(200) = 2.07, *p* = 0.040, d = 0.15. Please note that these results should be interpreted as specific for the set of images used in this study. They should not be broadly generalized to suggest that AI-generated images are universally preferred or consistently evoke more positive emotions compared to human-made artworks.

Because the participants could not discriminate between human- and AI-generated images, we did not perform separate analyses on the data from human- and AI-generated images (see also RQ2 as reported in the pre-registration). In Supplementary Materials we present also exploratory analyses which show that the above results concerning liking, positive emotions, and the participants inability to discriminate the source of the images generalizes across the included art styles (cubism, expressionism, impressionism, Japanese art, and abstract art).

### Background Variables

Gender did not have any statistically significant effects on liking the images, positive emotions, or on evaluating whether the images were generated by humans or AI. However, age interacted with beliefs about human vs AI -generated nature of the images, suggesting that the older the participants were and the more likely the images were rated as generated by AI, the less it was liked, B = − 0.003, 95% CI [− 0.004, − 0.002], *p* < 0.001, and the less positive emotions it evoked, B = − 0.004, 95% CI [− 0.005, − 0.003], *p* < 0.001. Age and category interacted in showing that, the older the participants were, the more likely they were to believe that the AI-generated images were made by humans, B = 0.173, 95% CI [0.100, 0.247], *p* < 0.001.

### Personality Variables

**Figure 1:**
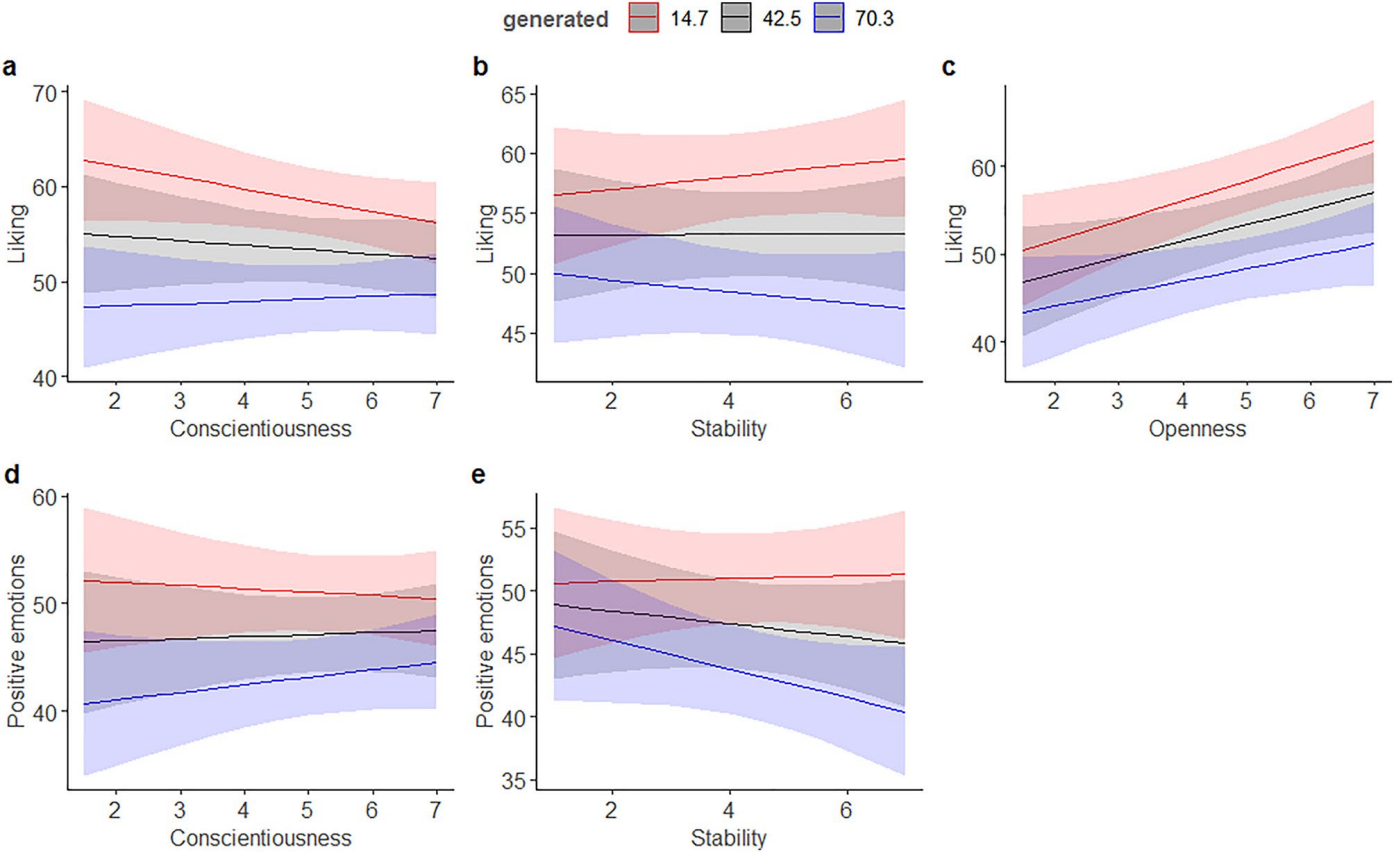
Liking and positive emotions evoked by the images as a function of personality variables and their interactions with the participants’ beliefs about whether the images were generated by humans or AI (0 = most likely human, 100 = most likely AI).

The models on liking and the origin of the images with personality traits, empathy, and beliefs about whether the images were generated by humans or AI as predictors (Table 3) showed that conscientiousness interacted with beliefs about the origin of the images such that the less conscientious the participants were, the larger the difference in liking and in positive emotions between images that were believed to be generated by humans as compared to images believed to be made by AI (Fig. 1a,d). The negative main effect showed for the variable “generated” (Table 3), indicates that the more strongly the images are believed to be generated by AI, the less they are liked and the less positive are the emotions toward them, point towards a general negative bias toward AI-generated artworks. In addition, the results for the trait emotional stability showed that the more emotionally stable the participants were, the more they liked and the more positive emotions were evoked when the images were believed to be generated by humans, compared to images believed to be produced by AI (Fig. 1b,e). Openness was related to liking the images and positivity of emotions: highly open persons liked the images and experienced positive emotions in general more than individuals on average. In addition, for highly open participants the difference in liking between images they believed were made by humans and those believed to be made by AI was larger than in participants on average (Fig. 1c).

**Table 3:** Liking and Positive emotions predicted by the big-five traits, empathy, and beliefs about whether the images were humans-made or AI-generated (0 = most likely human, 100 = most likely AI).

Predictors	Liking					Positive emotions				
	*B*	SE	95% CI	*t*	*p*	*B*	SE	95% CI	*t*	*p*
(Intercept)	53.27	1.77	49.80 to 56.73	30.15	** < 0.001**	47.17	1.78	43.67 to 50.67	26.44	** < 0.001**
Extraversion	− 0.15	0.98	− 2.07 to 1.77	− 0.15	0.878	0.47	1.06	− 1.61 to 2.55	0.44	0.661
Generated	− 4.99	0.23	− 5.44 to − 4.54	− 21.77	** < 0.001**	− 3.84	0.22	− 4.28 to − 3.41	− 17.26	** < 0.001**
Agreeableness	0.94	1.04	− 1.09 to 2.97	0.91	0.364	0.86	1.12	− 1.33 to 3.06	0.77	0.442
Conscientiousness	− 0.64	0.96	− 2.53 to 1.24	− 0.67	0.503	0.26	1.04	− 1.77 to 2.30	0.25	0.799
Openness	2.23	0.91	0.45 to 4.01	2.45	**0.014**	2.72	0.98	0.80 to 4.65	2.77	**0.006**
Stability	0.02	1.03	− 2.00 to 2.05	0.02	0.981	− 0.80	1.12	− 2.99 to 1.39	− 0.72	0.474
Empathy	− 0.22	1.03	− 2.25 to 1.80	− 0.21	0.831	0.04	1.12	− 2.15 to 2.23	0.03	0.974
Extraversion * generated	0.45	0.24	− 0.03 to 0.92	1.84	0.066	0.13	0.24	− 0.33 to 0.59	0.55	0.581
Generated * agreeableness	− 0.05	0.26	− 0.56 to 0.46	− 0.19	0.852	− 0.02	0.25	− 0.51 to 0.47	− 0.08	0.936
Generated * conscientiousness	0.99	0.23	0.54 to 1.43	4.35	** < 0.001**	0.69	0.22	0.26 to 1.12	3.12	**0.002**
Generated * openness	− 0.51	0.21	− 0.93 to − 0.09	− 2.38	**0.018**	− 0.40	0.21	− 0.81 to 0.01	− 1.92	0.056
Generated * stability	− 0.78	0.26	− 1.30 to − 0.27	− 2.97	**0.003**	− 0.98	0.26	− 1.49 to − 0.48	− 3.84	** < 0.001**
Generated * empathy	0.18	0.25	− 0.30 to 0.67	0.75	0.454	− 0.01	0.24	− 0.48 to 0.46	− 0.03	0.977

Significant values are in bold.

**Figure 2:**
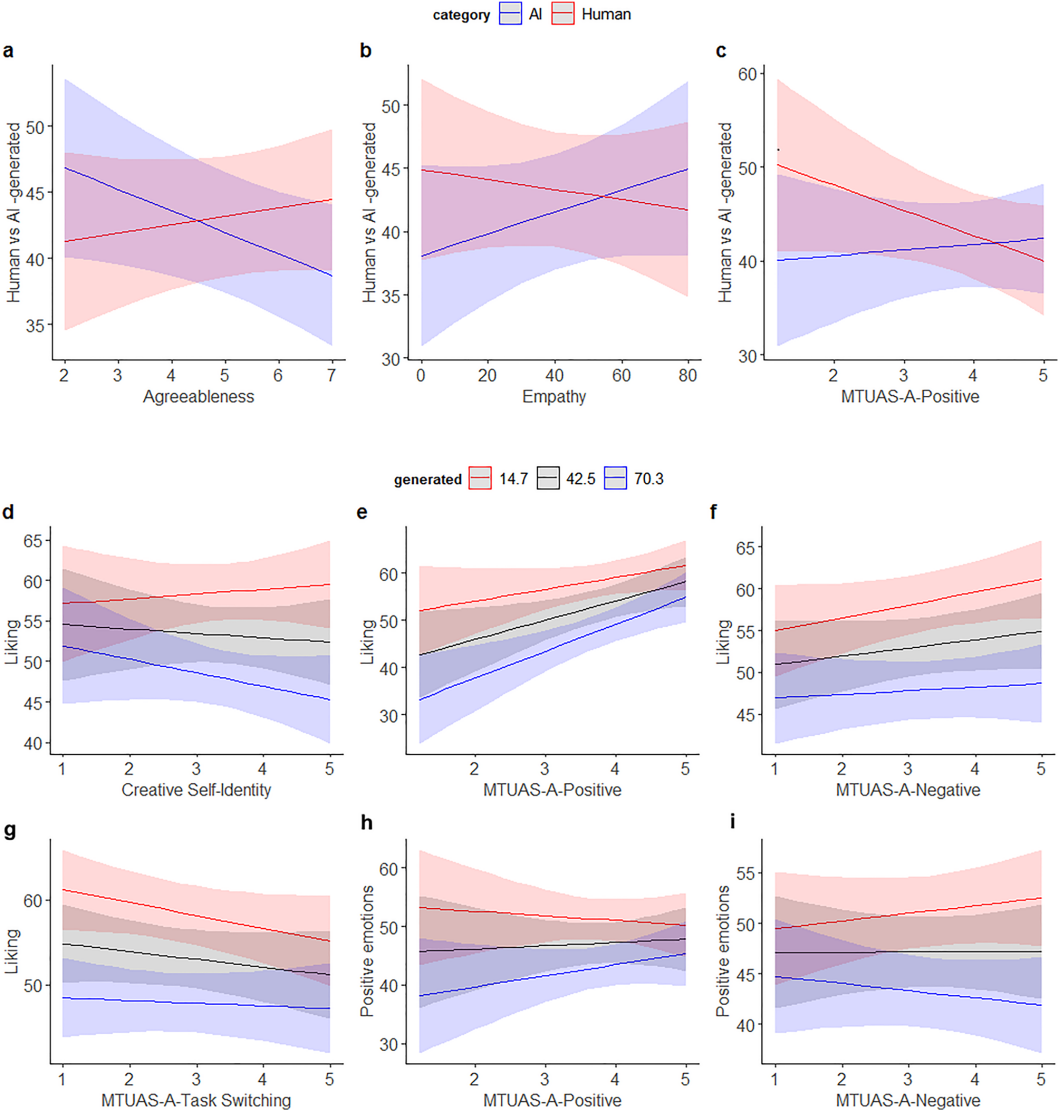
Top: Participants’ beliefs about whether the images were generated by humans or AI (0 = most likely human, 100 = most likely AI) as a Function of Agreeableness, Empathy, Positive Attitude Towards Media and Technology Usage, and the real category of the images (AI vs. Human). Bottom: liking and positive emotions elicited by the images as a function of Creative Self-efficacy and Personal Identity, Attitudes Towards Media and Technology Usage, and beliefs about whether the images were generated by humans or AI (0 = most likely human, 100 = most likely AI).

Next, we studied the effects of personality, empathy, and the real category of the images (human vs. AI -generated) on the beliefs about whether the images were generated by humans or AI (Table 4). The intercept was at 41.7, 95% CI [37.12, 46.20], on the scale from 0 (*most likely human*) to 100 (*most likely AI*), meaning that on average the AI-generated images (= the reference category) were rated as having been generated by humans. Agreeableness interacted with category, indicating that highly agreeable persons tended to rate human-made images as more likely to be made by AI than AI-generated images, whereas for individuals low in agreeableness the pattern was the opposite (Fig. 2a). In addition, empathy interacted with category, showing that the more empathic the persons, the better they could discriminate between AI- and human-generated (Fig. 2b), although even highly empathic persons could not discriminate between AI-made and human-made images in such way that their score would have been above 50 for AI-generated images (scores > 50 indicate that the image was attributed to AI, whereas scores < 50 indicate attribution to humans).

**Table 4:** Participants’ beliefs about whether the images were human-made or AI-generated (0 = most likely human, 100 = most likely AI) as a function of Big-Five traits and Empathy and the real category (AI vs. Human) of the images.

Predictors	Generated				
	*B*	SEr	95% CI	*t*	*p*
(Intercept)	41.66	2.32	37.12 to 46.20	17.99	** < 0.001**
Extraversion	− 1.53	0.91	− 3.32 to 0.25	− 1.69	0.092
Category [Human]	1.62	3.13	− 4.52 to 7.75	0.52	0.605
Agreeableness	− 1.95	0.96	− 3.83 to − 0.07	− 2.03	**0.042**
Conscientiousness	− 0.28	0.89	− 2.02 to 1.47	− 0.31	0.756
Openness	0.36	0.84	− 1.29 to 2.01	0.43	0.670
Stability	1.03	0.96	− 0.85 to 2.90	1.07	0.284
Empathy	1.20	0.96	− 0.68 to 3.08	1.25	0.210
Extraversion * category [Human]	0.07	0.63	− 1.17 to 1.30	0.11	0.914
Agreeableness * category [Human]	2.71	0.67	1.40 to 4.01	4.07	** < 0.001**
Category [Human] * conscientiousness	− 0.13	0.62	− 1.34 to 1.08	− 0.21	0.836
Category [Human] * openness	0.54	0.58	− 0.60 to 1.68	0.92	0.355
Category [Human] * stability	− 0.75	0.66	− 2.05 to 0.55	− 1.13	0.259
Category [Human] * empathy	− 1.75	0.66	− 3.05 to − 0.45	− 2.64	**0.008**

Significant values are in bold.

The next models examined liking and positive emotions as a function creative self-efficacy and personal identity, attitudes and usage of media and technology, interest and exposure to art, and beliefs about the source of the images (Table 5). As detected in previous analyses (see Table 3), also the current analysis reveals a significant trend for the variable "generated" (Table 5). Specifically, the results indicate that the stronger the belief that images are produced by AI, the less favorably they are perceived, and the more negative the emotional response towards them becomes, suggesting again a negative bias towards artworks generated by AI. Participants with high creative identity liked more than people on average the images they believed to be produced by humans, compared with those believed to be generated by AI (Fig. 2d). Participants with high positive attitude (Fig. 2e,h) towards media and technology liked and experienced positive emotions for images believed to be made by AI more than people on average, whereas participants with low negative attitude (Fig. 2f,i) showed the opposite pattern. However, participants with a highly positive attitude towards media and technology also generally liked images more than people on average. In addition, the higher the multitasking attitude in media and technology usage, the more the images were liked when they were believed to be human products, compared with images thought be produced by AI (Fig. 2g).

**Table 5:** Liking and positive emotions predicted by creative self-efficacy and personal identity, attitudes and usage of media and technology, interest and exposure to art, and beliefs about whether the image was generated by humans or AI.

Predictors	Liking					Positive emotions				
	*B*	SD	95% CI	*t*	*p*	*B*	SE	95% CI	*t*	*p*
(Intercept)	53.25	1.75	49.82 to 56.68	30.43	** < 0.001**	47.17	1.76	43.71 to 50.62	26.77	** < 0.001**
CSE	− 0.06	1.25	− 2.50 to 2.39	− 0.05	0.964	1.37	1.32	− 1.22 ro 3.96	1.03	0.301
Generated	− 5.31	0.23	− 5.76 to − 4.85	− 22.78	** < 0.001**	− 4.06	0.23	− 4.51 to − 3.62	− 17.94	** < 0.001**
CPI	− 0.59	1.44	− 3.42 to 2.23	− 0.41	0.680	− 0.89	1.53	− 3.89 to 2.10	− 0.59	0.558
MTUAS-A-P	− 2.71	1.10	− 4.86 to − 0.55	− 2.47	**0.014**	− 0.37	1.17	− 2.65 to 1.92	− 0.32	0.752
MTUAS-A-N	− 0.98	0.89	− 2.73 to 0.77	− 1.10	0.273	− 0.02	0.95	− 1.87 to 1.84	− 0.02	0.985
MTUAS-A- A	0.91	1.02	− 1.09 to 2.90	0.89	0.371	0.09	1.08	− 2.03 to 2.20	0.08	0.936
MTUAS-A-TS	0.92	0.89	− 0.82 to 2.66	1.03	0.302	− 0.02	0.94	− 1.87 to 1.83	− 0.02	0.986
ART Interest	1.50	1.35	− 1.15 to 4.16	1.11	0.267	3.87	1.44	1.05 to 6.69	2.69	**0.007**
ART Exposure	2.36	1.19	0.02 to 4.70	1.98	**0.048**	1.06	1.27	− 1.42 to 3.55	0.84	0.401
CSE * generated	0.53	0.29	− 0.04 to 1.11	1.81	0.070	− 0.17	0.29	− 0.73 to 0.39	− 0.60	0.545
Generated * CPI	− 1.26	0.35	− 1.94 to − 0.57	− 3.59	** < 0.001**	− 0.51	0.34	− 1.18 to 0.15	− 1.51	0.131
Generated * MTUAS-A-P	− 1.05	0.29	− 1.62 to − 0.49	− 3.65	** < 0.001**	− 0.89	0.28	− 1.44 to − 0.34	− 3.16	**0.002**
Generated * MTUAS-A-N	0.55	0.21	0.13 to 0.96	2.60	**0.009**	0.72	0.20	0.32 to 1.12	3.52	** < 0.001**
Generated * MTUAS-A-A	0.11	0.25	− 0.38 to 0.61	0.45	0.656	0.23	0.25	− 0.25 to 0.71	0.94	0.349
Generated * MTUAS-A-TS	− 0.60	0.22	− 1.03 to − 0.17	− 2.74	**0.006**	0.03	0.21	− 0.39 to 0.45	0.15	0.878
Generated * ART Interest	0.63	0.33	− 0.02 to 1.28	1.91	0.056	0.12	0.32	− 0.51 to 0.75	0.38	0.706
Generated * ART Exposure	0.19	0.31	− 0.41 to 0.80	0.63	0.529	0.33	0.30	− 0.25 to 0.92	1.12	0.263

*CSE* Creative Self-Efficacy, *CPI* Creative Personal Identity, *MTUAS-A-P* Media and Technology Usage and Attitudes Scale: positive attitude, *MTUAS-A-P* Media and Technology Usage and Attitudes Scale: negative attitude, *MTUAS-A-A* Media and Technology Usage and Attitudes Scale: anxiety, *MTUAS-A-TS* Media and Technology Usage and Attitudes Scale: Task switching.

Significant values are in bold.

Finally, we studied the participants’ believes on the generator of the images with the real category, creative self-efficacy and personal identity, the attitudes and usage of media and technology, and the real category (human- vs. AI-generated) of the images as predictors (Table 6). The only statistically significant effect was the category x MTUAS-A-P interaction Fig. 2c). This finding suggests that the more positive the attitude was towards media and technology, the less human-made images were believed to be AI-generated.

**Table 6:** Participants’ beliefs about whether the images were generated by humans or AI as a function of creative self-efficacy and personal identity, attitudes and usage of media and technology, and the real category of the images (human- vs. AI-generated).

Predictors	Generated				
	*B*	SE	95% CI	*t*	*p*
(Intercept)	41.66	2.32	37.12 to 46.20	17.99	** < 0.001**
CSE	0.70	1.18	− 1.61 to 3.02	0.60	0.551
Category [Human]	1.62	3.13	− 4.52 to 7.75	0.52	0.605
CPI	− 0.87	1.36	− 3.54 to 1.81	− 0.64	0.524
MTUAS-A-P	− 0.40	1.04	− 2.44 to 1.64	− 0.38	0.701
MTUAS-A-N	0.01	0.85	− 1.64 to 1.67	0.02	0.986
MTUAS-A-A	0.61	0.96	− 1.28 to 2.50	0.63	0.526
MTUAS-A-TS	− 0.37	0.84	− 2.02 to 1.29	− 0.43	0.665
ART interest	− 0.61	1.28	− 3.13 to 1.90	− 0.48	0.633
ART exposure	1.62	1.13	− 0.60 to 3.84	1.43	0.153
CSE * category [Human]	− 0.19	0.82	− 1.79 to 1.41	− 0.23	0.816
Category [Human] * CSI	1.71	0.94	− 0.14 to 3.57	1.82	0.069
Category [Human] * MTUAS-A- P	− 2.17	0.72	− 3.59 to 0.76	− 3.02	**0.003**
Category [Human] * MTUAS-A N	1.10	0.58	− 0.04 to 2.25	1.89	0.059
Category [Human] * MTUAS-A- A	0.27	0.67	− 1.04 to 1.58	0.41	0.685
Category [Human] * MTUAS-A-TS	− 0.11	0.58	− 1.25 to 1.04	− 0.18	0.855
Category [Human] * ART Interest	− 0.87	0.89	− 2.61 to 0.88	− 0.97	0.330
Category [Human] * ART Exposure	− 0.89	0.78	− 2.42 to 0.64	− 1.14	0.256

*CSE* Creative Self-Efficacy, *CPI* Creative Personal Identity, *MTUAS-A-P* Media and Technology Usage and Attitudes Scale: positive attitude, *MTUAS-A-P* Media and Technology Usage and Attitudes Scale: negative attitude, *MTUAS-A-A* Media and Technology Usage and Attitudes Scale: anxiety, *MTUAS-A-TS* Media and Technology Usage and Attitudes Scale: Task switching.

Significant values are in bold.

## Discussion

The present study primarily aimed to investigate how individual factors might influence the way people perceive and evaluate artworks, particularly based on their beliefs about the origins of these images—whether they are human-made or AI-generated (RQ1). Exploratorily, drawing from the pre-registered data analysis plan, the study aimed to evaluate the participants' ability to effectively differentiate between the two image categories (Human-made or AI-generated) and identify which individual characteristics might predict their success in such differentiation (RQ2). Furthermore the study attempted to determine if participants had a negative bias toward AI-generated artworks (RQ2).

In agreement with our hypothesis (H1b), participants with a high creative identity demonstrated a stronger liking for images they believed to be produced by humans, as opposed to those they thought were generated by AI. This inclination may stem from the perception that AI-generated art lacks the authenticity or expressiveness typically associated with human-created art, resulting in a negative bias towards these creations^54^. Furthermore, individuals with a strong attachment to art may view AI-generated art as a threat to the traditional artistic process, which is deeply rooted in human creativity and expression^61^. The emergence of AI-generated art challenges the conventional understanding of art as an exclusively human endeavor, possibly provoking feelings of skepticism or disapproval from those with a high creative identity. This reaction could further exacerbate the negative bias towards AI-generated images, as these individuals may perceive AI art as a lesser form of creativity, lacking the emotional depth and unique perspective offered by human artists in an anthropocentric perspective^53^. Additionally, this negative bias might be influenced by concerns about the potential implications of AI-generated art on the art world, such as the devaluation of human-created art and the loss of artistic jobs as AI technology becomes more cost-effective, fast, and accessible^62^. Furthermore, some people may think AI-art is unethical due to the ongoing dispute about the use of copyrighted art images in the AI algorithm trainings^63–65^. As a result, individuals with a high creative identity may not only appreciate human-generated art more but also actively resist the adoption of AI-generated art due to their emotional investment in the traditional artistic process. However, contrary to our hypothesis, creative self-efficacy did not show an effect on liking the images depending on their source attribution. Against our hypothesis (H1a,c), empathy and art exposure/interest did not influence how the participants perceived the images in relation to how much they believed to be human-made or AI-generated.

In line with our hypothesis (H2), individual relationship with technology predicts a more positive bias towards AI-generated art, with people displaying higher scores of positive attitudes toward technology liking more and perceiving more positive emotions for images that are perceived more likely to be AI generated rather than human-generated, while the opposite effect was shown for negative attitude toward technology. Also, the higher the task-switching/multitasking technology attitude, the smaller was the difference between liking the images perceived as AI-generated, compared with those perceived as human-generated. This latter finding can be related to the fact that users who prefer multitasking are more used to technology and previous research show preference for task switching and positive attitude toward technology to be positively related^66^.

Contrary to our hypothesis (H3), we found that openness, a personality trait generally associated with an interest in art and creativity^67–69^, positively predicted liking of images that were perceived as AI-generated. Furthermore, participants scoring high in open to experience liked the images more and experienced more positive emotions in general compared to individuals on average. This finding points towards an alternative hypothesis compared to our pre-registered one. Open to experience individuals may be more accepting towards the products of new technologies than people on average and may be more likely to embrace the potential of new technologies^70–72^. These results are consistent with some previous studies that have found a positive association between openness and appreciation of various forms of innovative art^73^, or attitude to experience art with the support of modern technologies^74,75^.

In our exploratory analysis of the other big-five personality traits, we found that conscientiousness and emotional stability influenced the perception of the origin of the images. Less conscientious participants showed a larger difference in liking and positive emotions between images believed to be generated by humans than those believed to be made by AI. In addition, more emotionally stable participants liked images they believed were human-generated more and experienced more positive emotions in response to them compared to images they believed to be AI-generated. These results suggest that personality traits beyond openness may also play a role in the perception and evaluation of AI-generated art, reflecting the multifaceted nature of individual differences in aesthetic experiences^76^.

Initial analyses showed that the study participants could not reliably discriminate between human and AI-generated images, which suggests that AI-generated art has become highly human-like, consistent with the increasing sophistication of AI technologies^29,77,78^. Follow-up exploratory analyses found that highly empathic people may be more sensitive than people on average in discriminating between AI- and human-generated art. This result may be related to previous findings showing a relationship between empathy and the appreciation of emotional content in art^79^. It also raises the possibility that individuals may be more attuned to the social traces of human creativity in the images^51^. However, our results shows that these cues are not sufficient to enable accurate attribution of the images' origin even in highly empathetic individuals, as even these individuals were unable to discriminate human-made from AI-generated images above chance level.

The exploratory analyses performed to understand a possible negative bias toward AI-generated art, revealed firstly that participants liked AI-generated images more than our selection of human-made images and experienced more positive emotions with AI-generated images (considering the true sources of the images). These findings confirm other evidence that has shown that AI-generated art is perceived as evoking emotional reactions and that AI art has technologically reached a high level when it comes to quality and ability to communicate emotions^80,81^. Nevertheless, the observation that our participants rated the AI-generated artworks more positively compared to the human-made ones, yet rated artworks they subjectively perceived as AI-generated more negatively, effectively determined the presence of a negative bias in the evaluation of artworks believed to be AI-generated. Although the artworks created by AI were liked more and evoked more positive emotions compared to the selection of human-made artworks in our study, the opposite trend emerged when considering the participants' subjective ratings. When participants perceived images as AI-generated, they tended to rate them as being of lower quality and eliciting lower level of positive emotions. These findings support the notion, in line with previous literature^28^ and with our expectations, that humans exhibit a negative bias towards artworks generated by AI. Such bias may extend behind artworks into other AI-generated outputs. Future studies should investigate if such bias is universal for AI-generated products, and what can be done to mitigate such bias, especially in the context of AI applications where AI could critically assist humans and increase human performance.

Several limitations should be considered when interpreting our findings. First, our sample consisted mostly of highly educated participants, which may limit the generalizability of our results in the other age groups. Second, the experimental design relied on a relatively small set of images representing five art styles, which does not capture the full diversity of human- and AI-generated art. Future research could address these limitations by employing a more diverse sample and examining a broader range of art styles.

Using a different set of stimuli potentially influences the reported finding wherein humans were unable to accurately identify the true source of the images. It is plausible that certain types of artworks exhibit characteristics widely recognized as indicative of human creativity, thereby enabling discrimination between human-made and AI-generated artworks. Nevertheless, the observation that our participants rated the AI-generated artworks more positively compared to the human-made ones, yet rated artworks they subjectively perceived as AI-generated more negatively, effectively pinpointed the presence of an negative bias in the evaluation of artworks believed to be AI-generated.

The study's sample was gathered from individuals volunteering for research tasks through the online platform Prolific, which may result in a sample primarily consisting of individuals who possess greater IT proficiency than the average population. Although Prolific users are generally known to provide high-quality data^82^, online study participants may exhibit less motivation than, for instance, students completing surveys on campus. Nonetheless, the implementation of attention-check control questions should have restricted or eliminated participants who answered randomly or without carefully reading each item of the online survey and experiment.

Further research is necessary to examine the intricate relationship between individual factors and the perception and evaluation of AI-generated art in various contexts and across a diverse range of art styles. The inability of participants to consistently differentiate between human- and AI-generated images indicates that AI has achieved a level of refinement capable of creating art resembling human-produced works. This raises questions about the intrinsic value and distinctiveness of human-generated art and the potential for AI-generated art to impact and even transform the human perception of creative work.

Moreover, we recommend conducting a study repeating the current experimental design in a few years. This would enable us to test whether attitudes towards AI-generated art evolved over time, potentially due to increased exposure and familiarity with AI-generated artworks or shifts in cultural or aesthetic norms. It could also provide valuable insights into whether the traits that influence the perception and evaluation of AI-generated art today continue to play a role in the future, or whether these traits play a role only during the early contact to the new technology.

## Conclusions

In conclusion, our research offers novel perspectives on the perception and assessment of AI-generated art, enriching the overall understanding of machine-created artwork. Our findings emphasize the importance of individual factors, such as personality traits and attitudes towards technology, in the perception and evaluation of stimuli subjectively perceived as human-made or AI-generated. We believe that such findings may be generalizable to other AI-generated contents.

Our results support the idea that humans may devaluate AI-generated artworks. This is possibly due to a cognitive bias (as e.g., proposed by Anthropocentrism theory^53^). In fact, the participants of our study perceived AI-generated artworks as generally more likeable and eliciting a higher degree of positive emotions compared to human-made ones. However, when the subjective belief of the source of the artworks was considered, the participants preferred what they believed to be a human-made artwork and not an AI-generated one.

## Method

The study hypotheses, methodology, and data analysis were developed before the data collection, and pre-registered using OSF. The pre-registration can be found at this link**:** https://osf.io/8n3bv?view_only=16b900f1c0fe440b82a58c6a6b5498f7. Deviations from the pre-registration are directly mentioned in the article text or defined as exploratory.

### Participants

A group consisting of 206 adults from the UK population was recruited online through the website Prolific to participate in the study. This convenience sample was balanced for gender and included individuals who self-reported having normal or corrected-to-normal vision, and who used a PC or laptop with a physical keyboard. Participants completed an experiment and a battery of questionnaires, implemented using Psyktoolkit^83,84^. The battery contained four attention check questions, such as selecting the highest or lowest value for a given item. Data from those who failed to answer one or more attention checks correctly was excluded from the final sample. In total, data from five participants was excluded due to incorrect responses to the attention check questions.

The experiment was performed in accordance with relevant national and international guidelines and regulations for research ethics and data privacy. All participants confirmed their willingness to participate in the experiment and provided informed consent after reading and accepting the terms prior to the start of the survey. All experimental protocols were approved by the University of Bergen and were accomplished according to the guidelines of Norwegian Agency for Shared Services in Education and Research regarding data privacy and processing.

Generally, 10 to 20 participants per predictor are considered an appropriate number for regression-based analyses. On this basis, we estimated that around 200 participants will be an appropriate number for analyses having more than 10 but less than 20 predictors (i.e., fixed effects in linear mixed-effect analyses). The final sample consisted of 201 participants (99 females, 100 males, and 2 other). Their mean age was 41.0 years (SD = 14.6, min = 18, max = 76). One-hundred and twenty-one of them (62%) had a university degree, 77 had a high school degree (38%), 1 had a middle school and 2 had an elementary school degree.

### Materials

In this study, a total of 40 images were utilized, with 20 generated using generative AI text-to-image prompts and 20 sourced from the internet as human-created art pieces. The AI-generated images were produced by Midjourney v. 4 AI text-to image tool, using prompts related to five distinct art styles that were chosen according to our discretion. The selected styles were Expressionism, Impressionism, Cubism, Abstract, and Traditional Japanese art (also prompted as “Edo” art). The prompt was repeated until acceptable quality of the images was obtained (e.g., avoiding produced images with distorted figures or parts written in them). The prompts used were of the format, e.g., "Impressionism landscape, painting." To select human-made art pieces, the study authors chose copyright-free (CC) artworks (both paintings for historical art styles, and digital art for abstract art styles) using queries in Google Images and using research words like those provided as prompt to the AI. This approach was intended to ensure that the human-made stimuli exhibited comparable general content to their AI-generated counterparts. Please note that the human-made images were selected solely according to the discretion of the study authors, without considering aesthetic characteristics, therefore they should not be meant as a direct and fair comparison to the AI-generated images used in the study. Human-made art pieces were subsequently cropped to maintain consistency with the square format generated by Midjourney v. 4. During this process, the primary goal was to preserve the main scene of each artwork. As the digital quality of the human-made images, which were obtained through photography or scanning processes, varied and was generally lower compared to the AI-generated images, random white noise was introduced to attempt to match the graphic quality across all images (or at least reduce the difference). This was done as the AI-generated art, being optimized to be displayed on screen, looks in general sharper and brighter in colors compared to human-made art, that is instead generally painted on canvas and digitalized using photos. This manipulation was designed to equalize the digital quality of both AI-generated and human-made images. By doing so, it aimed to facilitate a more accurate and reliable comparison between these two distinct types of visual stimuli.

### Scales

The following list reports the scales that were used in the study. The scales are presented in order as they were presented in the original online survey.

The Ten-Item Personality Inventory (TIPI)^85^ is a brief self-report measure designed to assess the Big-Five personality dimensions: extraversion, agreeableness, conscientiousness, emotional stability (the reverse of neuroticism), and openness to experience. The TIPI has been proposed as a time-efficient alternative to longer Big-Five personality assessments. The TIPI consists of 10 statements, with each dimension represented by two items—one positively worded and the other reverse-coded. Participants rate themselves on a 7-point Likert scale ranging from “Strongly Disagree” to “Strongly Agree.” Despite its brevity, the TIPI demonstrates acceptable levels of reliability and validity when compared to more extensive personality measures^86,87^. For its brevity, the scale is particularly adequate for long surveys and online studies.

The Empathy Quotient (EQ): self-report measure designed to assess empathy levels in adults. The EQ scale consists of 40-item^88^ as a comprehensive tool to measure cognitive and affective empathy across three domains: cognitive empathy, emotional reactivity, and social skills. Participants rate themselves on a 4-point Likert scale, ranging from “Strongly Disagree” to “Strongly Agree.” The EQ has demonstrated satisfactory reliability and validity^89,90^ and in the present data its Cronbach’s α was 0.91. It has been widely used in research on social cognition^91^, and related areas such as autism spectrum conditions^92^.

The Short Scale of Creative Self (SSCS): this scale^93,94^ is a self-report measure developed to assess trait-like creative self-efficacy (CSE) and creative personal identity (CPI). CPI refers to the belief that creativity is a crucial aspect of an individual's self-description. The SSCS consists of 11 items, with six items measuring CSE and five items measuring CPI. Participants rate their agreement with each statement on a 5-point Likert scale. While CSE and CPI are often studied together, the SSCS subscales for CSE and CPI can also be used independently^95^. Cronbach’s α was 0.84 for CSE and 0.94 for CPI in the present data.

The Media and Technology Usage and Attitudes Scale (MTUAS): The MTUAS scale^96^ is a self-report measure developed to assess individuals' usage patterns and attitudes towards various forms of media and technology. The MTUAS examines multiple dimensions of media and technology use, including daily usage time, multitasking, and anxiety or negative feelings related to media and technology. The full MTUAS is composed of 44 items covering 14 subscales, such as smartphone usage, social networking, texting, email, video watching, and video gaming, among others. However, in the present study only 16 items related to attitudes towards technology (positive, negative, anxiety, and task-switching) were used. Participants rated their agreement with each of the proposed statements on a 5-point Likert scale (strongly agree, agree, neither agree nor disagree, disagree, strongly disagree). The Cronbach’s α in the present data was 0.80 for positive attitude, 0.77 for negative attitude, 0.85 for anxiety, and 0.89 for task-switching. The scale has been extensively used in the field of human computer interaction^97^.

The Vienna Art Interest and Art Knowledge Questionnaire (VAIAK). The questionnaire^98^ is a self-report measure designed to assess both art interest and art knowledge in individuals. The VAIAK was developed to provide a unified and validated instrument for exploring people's engagement with and understanding of visual art across various research contexts, such as psychology, art history, and education. The VAIAK consists of 24 items divided into two subscales: Art Interest (11 items) and Art Knowledge (10 items). However, only the items regarding art interest were used in the present study. Participants rated their agreement with the Art Interest statements (7 items) on a 7-point Likert scale (from not at all to completely), or stating how often they engaged in activities related to art (e.g., visiting museums and art galleries) (4 items), using a 7-point scale (less than once a year, once per year, once per half-year, once every three months, once per month, once every fortnight, once a week or more often). In our analyses these two groups of questions of the VAIAK Art Interest were treated as two different sub-scales (labeled in our study as art interest and art exposure). In the present data, the Cronbach’s α for Art interest was 0.94 and for Art exposure 0.76.

### Procedure

Upon obtaining informed consent and gathering background details such as age, gender, and level of education, the participants started the image-presentation stage of the study, which was then succeeded by the questionnaire phase. In the image-presentation phase of the study, the participants were presented with the images in random sequence (in total 40 images, 20 AI-generated and 20 human-made). Please note that during the image presentation, the participants were not aware of the true origin of the images. The participants were instructed to answer four questions relative to the image that was shown, using a sliding bar from 0 to 100. The proposed questions were: How much do you like the image? (from 0: not at all, to 100: very much), Does the image evoke positive emotions? (from 0: not at all, to 100: very much); do you think that the image was made by a human or generated by artificial intelligence? (from 0: most likely made by a human to 100: most likely generated by artificial intelligence); Have you seen this image before? (online, in a book, in a museum etc.), from (0: I am sure I have never seen it, to 100: I am sure I have seen it before). There was no time limit for the participants to give the responses. After all the images were shown and response collected, the study proceeded to the questionnaire phase. In this phase, the participants had to complete a series of questionnaires about their personality, attitudes, relationship with art, and relationship with technology. The questionnaires used were (in this order): TIPI, EQ, SSCS, MTUAS (only attitude sub-scale), and the VAIAK (only art interest sub-scale).

### Statistical analyses

Initial analyses found that our participants were not able to discriminate between human-made and AI-generated images. Therefore—following one of the planned options in the analysis plan in the pre-registration—we decided to not to analyze the individual predictors separately for (objective source) AI-generated and Human-made images, and to analyze all the images together in the same model. Consequently, the research question number 2 reported in the pre-registration was not directly assessed. Recognizing from these analyses that the source of the images was in fact “blinded” to the participants, further exploratory analyses were performed. These exploratory analyses were aimed to understand the level of appreciation of the image categories in general (objective sources) and then to assess the level of appreciation for the images in function of the subjective perception of the participants perceiving them as human-made or AI-generated. These analyses were performed to assess a possible negative bias toward AI-generated artworks. Further exploratory analyses were performed to understand if some individual factors predicted the participant’s ability to effectively discriminate human-made from AI-generated images.

The statistical analyses were conducted with jamovi^99^ for descriptive statistics and correlations and the linear mixed-effect analyses were performed with R. First, we computed descriptive statistics and Pearson’s correlations, examining intercorrelations between the predictor variables and between the predictor variables and the dependent variables. Then paired samples t-tests were used to explored whether AI- or human-generated images were perceived as more likeable, whether AI- or human-generated images evoked more positive emotions, and whether the participants can discriminate between AI- and human-generated images. The likeability variables were normally distributed (Shapiro–Wilk test, *p* > 0.05), while the other variables were slightly skewed to the left (Shapiro–Wilk test, *p* < 0.05). We used t-test as it can be relatively robust to small violations of normality assumptions, especially when the skewness is in the same direction and the sample size is relatively large.

The linear mixed-effect models examined how individual factors predict the ratings were performed with linear mixed effect analyses on item level data. Note that in the linear mixed effect analyses that we report the coefficient B refers to unstandardized coefficients. Because the t-tests indicated that the participants could not discriminate between human- and AI-generated images, the following analyses were performed on all images without separating human-generated and AI-generated images, as preregistered. Separate models were performed for each dependent variable (1) liking of the images, (2) positivity of emotions, and (3) perception of image source: human- vs. AI-generated. These analyses were performed for three sets of predictor variables, the first involving age and gender, the second involving personality (TIPI Big-Five and EQ), and the third one involving MTUAS, VAIAK, and SSCS.

In the first set of linear mixed-effect model, we examined the effects of background variables on liking the images and on positive emotions towards them. Most of the participants (98%) had a high school or university degree so that they were mostly relatively highly educated and therefore education level was not included as a predictor in the statistical models. Thus, the models included age, gender (female, male; n = 199 as two participants did not identify as male or female) and their interactions with beliefs about whether the images were generated by humans or AI as fixed effects. The random effects were the random intercepts for the participants and for the items (i.e., each specific image) here and in all the models that follow.

After examining the background variables, linear mixed effect models were performed on the personality variables by entering the Big-Five traits and empathy as the fixed effects as well as their interactions with the predictor variable concerning participants' beliefs on the image source (human- vs. AI-generated). The liking and positivity of emotions were dependent variables in separate models, and the random intercepts for the participants and for the items (i.e., images) served as the random effects. The model including the subjective beliefs on the image source (human- vs. AI-generated) as the dependent variable involved the personality variables and the objective category (human- vs AI-generated) with its interactions with the personality variables as the fixed effects.

The final set of linear mixed effect models involved as fixed effects the scores from the other than personality scales (MTUAS, VAIAK, SSCS) and their interactions with the predictor variable concerning participants' beliefs on the image source (human- vs. AI-generated). Deviating from the preregistered analysis plan, we included in these analyses all the measured sub-scale scores from the MTUAS (attitude component), VAIAK, and SSCS inventories, because unexpectedly there was no problems with collinearity (VIF-values < 5), despite the strong correlations between scales. Otherwise, the structure of the models was identical to the models performed on the personality variables. We did not have explicit hypotheses for the sub-scales of MTUAS (attitude component) regarding technology anxiety and multitasking, however as we collect this data in the context of MTUAS-attitude, we included these sub-scales in the statistical models, and we report the results for these scale in the article for completeness.

### Supplementary Information

Supplementary Information.

## Data Availability

The dataset used for the analyses reported in the study is available in osf.io at the following link: https://osf.io/ugfps/?view_only=0d78aefec02549f4afc2b14fa635cb0a.
