# Supplementary material for: Understanding how personality traits, experiences, and attitudes shape negative bias toward AI-generated artworks
Source: Sci Rep. 2024 Feb 19;14:4113. doi: 10.1038/s41598-024-54294-4 (PMC10876601; doi:10.1038/s41598-024-54294-4)
Supplement: Supplementary file 1 — Supplementary Information. [file 41598_2024_54294_MOESM1_ESM.pdf]

Supplementary materials for the manuscript: “Exploring the Impact of Individual Factors, Experiences, and Attitudes on Perception and Evaluation of AI-Generated Art”

Table S1

Intercorrelations Between Personality Measures

	Extra-version	Agree-ableness	Conscien-tiousness	Stability	Openness
Agreeableness	0.010	—			
Conscientiousness	0.039	0.280 ***	—		
Stability	0.303 ***	0.321 ***	0.419 ***	—	
Openness	0.316 ***	0.062	-0.040	0.068	—
Empathy	0.255 ***	0.461 ***	0.208 **	0.116	0.246 ***

Note. * p < .05, ** p < .01, *** p < .001

Table S2

Intercorrelations Between Creative Self-Efficacy, Personal Identity, Attitudes and Usage of Media and Technology, and Interest and Exposure to Art

	CSE	CPI	MTUAS-A-P	MTUAS-A-N	MTUAS-A-A	MTUAS-A-TS	ART Interest
CPI	0.699 ***	—					
MTUAS-A-P	-0.112	0.051	—				
MTUAS-A-N	0.080	0.007	-0.365 ***	—			
MTUAS-A-A	0.043	-0.036	0.513 ***	-0.150*	—		
MTUAS-A-TS	0.000	-0.175 *	0.182 **	-0.031	0.272***	—	
ART Interest	0.358 ***	0.616 ***	0.170 *	-0.008	-0.026	-0.104	—
ART Exposure	0.228 **	0.450 ***	0.194 **	-0.025	0.071	-0.058	0.713 ***

Note. * p < .05, ** p < .01, *** p < .001; MTUAS-A-P = Media and Technology Usage and Attitudes Scale: positive attitude, MTUAS-A-P = Media and Technology Usage and Attitudes Scale: negative attitude, MTUAS-A-A = Media and Technology Usage and Attitudes Scale: anxiety, MTUAS-A-TS = Media and Technology Usage and Attitudes Scale: Task switching

Table S3

Pearson's Correlations Between the Predictors and the Dependent Variables(n = 201)

Predictor	Liking	Pos.emotions	Generated
Extraversion	0.054	0.098	-0.100
Agreeableness	0.073	0.073	-0.027
Conscientiousness	-0.038	0.011	-0.024
Stability	0.012	-0.003	-0.005
Openness	0.178*	0.218**	0.025
Empathy	0.052	0.093	-0.017
CSE	0.061	0.181*	0.048
CSI	0.079	0.224**	0.037
MTUAS-A-P	-0.091	0.025	0.075
MTUAS-A-N	-0.035	-0.002	0.024
MTUAS-A-A	-0.013	-0.013	0.101
MTUAS-A-TS	0.035	-0.034	-0.006
ART Interest	0.188**	0.350***	0.013
ART Exposure	0.212**	0.279***	0.073

Note. * p < .05, ** p < .01, *** p < .001; MTUAS-A-P = Media and Technology Usage and Attitudes Scale: positive attitude, MTUAS-A-P = Media and Technology Usage and Attitudes Scale: negative attitude, MTUAS-A-A = Media and Technology Usage and Attitudes Scale: anxiety, MTUAS-A-TS = Media and Technology Usage and Attitudes Scale: Task switching

Figure S1and Table S4 present the results from models in which the fixed effects were the art style, the participants' beliefs about whether the images were generated by humans or AI, and their interaction. The results show that the impressionist and Japanese art styles were the most liked and they elicited the most positive emotions, and more so when the real source of the images was AI. On the other hand, the impressionist and Japanese art styles were mostly believed to be products of humans.

Figure S1

Liking, Positive Emotions, and Participants' Beliefs About Whether the Images Were Generated by Humans or AI as a Function of Art Style and the Real Category of the Images (AI vs. Human)

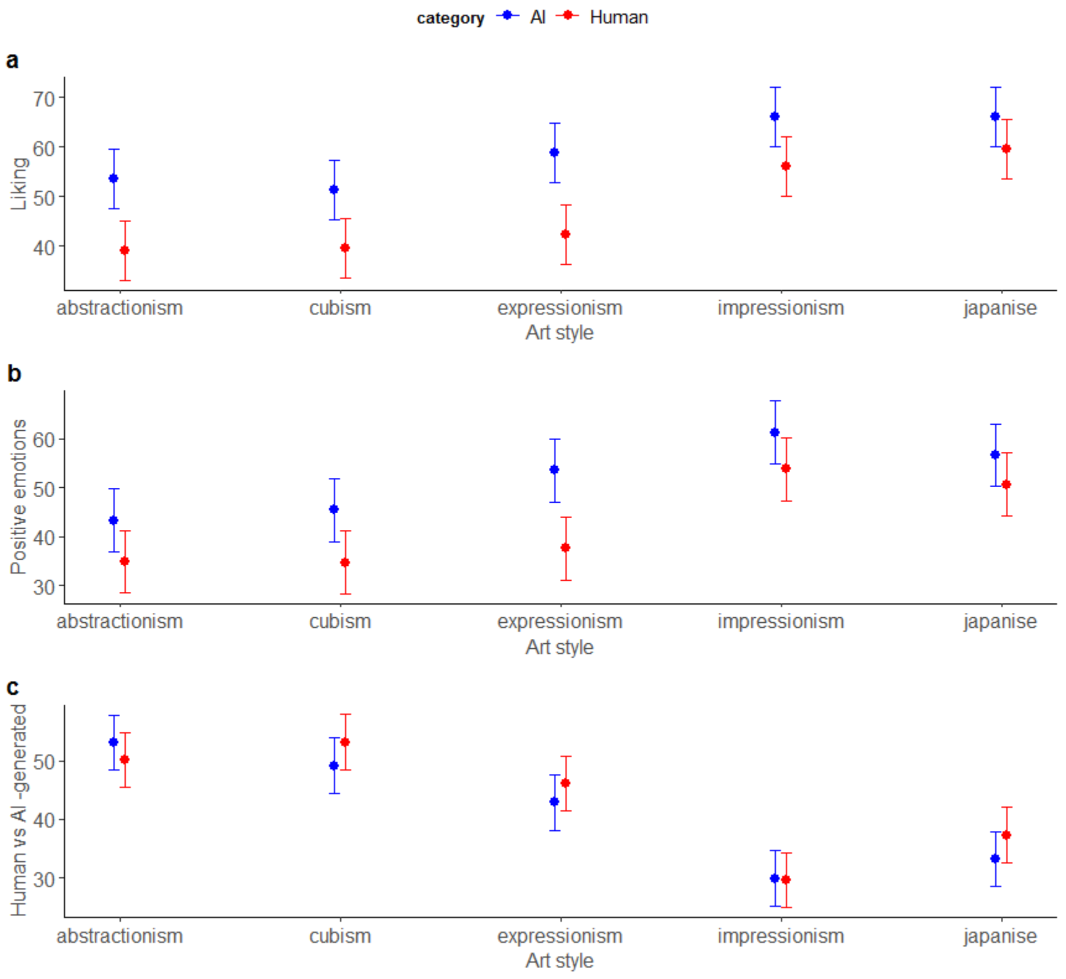

Table S4

Liking, Positive Emotions, and Participants' Beliefs about Whether the Images Were Generated by Humans or AI as a Function of Art Style and the Real Category of the Images (AI vs. Human)

	Liking			Positive Emotions			Generated		
Predictors	*B*	*95% CI*	*p*	*B*	*95% CI*	*p*	*B*	*CI*	*p*
(Intercept)	53.60	47.62 – 59.57	**<0.001**	43.25	36.81 – 49.69	**<0.001**	53.09	48.39 – 57.79	**<0.001**
category [Human]	-14.46	-22.59 – -6.34	**<0.001**	-8.39	-17.14 – 0.36	0.060	-3.00	-9.37 – 3.37	0.356
style [cubism]	-2.18	-10.30 – 5.95	0.600	2.21	-6.55 – 10.96	0.621	-3.90	-10.27 – 2.47	0.230
style [expressionism]	5.16	-2.97 – 13.29	0.213	10.27	1.52 – 19.02	**0.021**	-10.25	-16.62 – -3.87	**0.002**
style [impressionism]	12.46	4.33 – 20.58	**0.003**	18.03	9.28 – 26.78	**<0.001**	-23.19	-29.56 – -16.82	**<0.001**
style [japanese]	12.36	4.23 – 20.49	**0.003**	13.36	4.61 – 22.11	**0.003**	-19.82	-26.19 – -13.45	**<0.001**
category [Human] * style [cubism]	2.57	-8.92 – 14.06	0.661	-2.37	-14.75 – 10.01	0.708	7.01	-1.99 – 16.02	0.127
category [Human] * style [expressionism]	-2.01	-13.51 – 9.48	0.731	-7.56	-19.94 – 4.82	0.231	6.29	-2.72 – 15.30	0.171
category [Human] * style [impressionism]	4.57	-6.92 – 16.07	0.435	0.84	-11.54 – 13.22	0.894	2.73	-6.28 – 11.74	0.552
category [Human] * style [japanese]	7.99	-3.51 – 19.48	0.173	2.36	-10.02 – 14.74	0.709	7.04	-1.97 – 16.05	0.126
